# Exoworkathlon: A prospective study approach for the evaluation of industrial exoskeletons

**DOI:** 10.1017/wtc.2022.17

**Published:** 2022-09-19

**Authors:** Verena Kopp, Mirjam Holl, Marco Schalk, Urban Daub, Enrique Bances, Braulio García, Ines Schalk, Jörg Siegert, Urs Schneider

**Affiliations:** 1 Fraunhofer Institute for Manufacturing Engineering and Automation IPA, Department Biomechatronic Systems, Nobelstraße 12, 70569 Stuttgart, Germany; 2 Institute of Industrial Manufacturing and Management IFF, University of Stuttgart, Allmandring 35, 70569 Stuttgart, Germany

**Keywords:** exoskeletons’ evaluation, occupational health, realistic working tasks

## Abstract

Industrial exoskeletons have recently gained importance as ergonomic interventions for physically demanding work activities. The growing demand for exoskeletons is leading to a need for new knowledge on the effectiveness of these systems. The Exoworkathlon, as a prospective study approach, aims to assess exoskeletons in realistic use cases and to evaluate them neutrally in their entirety. For this purpose, a first set of four realistic Parcours was developed with experts from relevant industries, the German Social Accident Insurance, and the Federal Institute for Occupational Safety and Health. In addition, a set of ratings was defined to assess subjective user feedback, work quality, and objective physiological parameters. Exoworkathlon aims to bring together developers, researchers, and end-users, strengthen collaborative exchanges, and promote a platform for the prospective holistic data collection for exoskeleton evaluation. In this article, the focus is on the background and methodology of Exoworkathlon.

## 1. Introduction

Industrial exoskeletons are assistive tools for heavy physical work with a still young history of use. There are increasing examples of their application in industrial tasks such as box picking in logistics, car body assembly, manual welding, or construction (Linner et al., 2018; Crea et al., 2021; Marinov, 2021; Schmalz et al., 2021; Pacifico et al., 2022). The main reasons for using exoskeletons are high-stress levels for workers, which are difficult to eliminate by technical or organizational measures (Daub, 2017). Especially musculoskeletal disorders (MSDs) of the spine and shoulder are relevant occupational illnesses closely related to heavy physical work (Schneider et al., 2010; EU-OSHA, 2020). Various studies have demonstrated corresponding effects on biomechanic or metabolic parameters by wearing an exoskeleton during physically demanding activity. Other frequently investigated parameters are subjectively perceived effort, exoskeleton comfort, and usability (Kim and Nussbaum, 2019; Alemi et al., 2020; Koopman et al., 2020; Crea et al., 2021).

These kinds of studies are mainly laboratory studies and tasks vary broadly from static to dynamic with one or many repetitions. Less often, tasks were set that more closely resembled real work situations. However, since the effectiveness of exoskeletons depends on the use cases, studies should be carried out in dedicated experiments (Crea et al., 2021).

In order to generate valuable results and perform an objective analysis of the effects of exoskeletons in a real work environment, evaluation methods are essential (Masood et al., 2019; Planas-Lara et al., 2022). Most current studies lack larger sample sizes or standardized parameters (Grazi et al., 2019; Crea et al., 2021; Hoffmann et al., 2022; Sposito et al., 2022). In this respect, the EUROBENCH project has already taken the initiative and developed a method and framework for companies to test the performance of their robotic prototypes in a standardized way for benchmarking their exoskeletons in a unified manner. The goal is to increase the reproducibility and comparability of robotic systems (Torricelli and Pons, 2019; EUROBENCH, 2022).

From our point of view, however, the exoskeleton branch is still very young and consists mainly of startups. Therefore, it is challenging for individual exoskeleton companies to generate valuable and sufficiently large amounts of data that can show the effects of exoskeletons urgently required by potential users (Schalk et al., 2021). Previous study designs such as EUROBENCH are based on investigating or benchmarking the effects of single exoskeletons in an isolated activity and are mostly conducted with laypersons as subjects. Exoworkathlon differs from this in several ways. It does not limit the analysis to a single system but evaluates the effectiveness of exoskeletons in their entirety, independent of the manufacturer. In addition, the tasks are embedded in a realistically simulated work process and performed by young experts from the respective work areas, not by laypersons. Furthermore, it aims to evaluate the subjective user feedback, the muscle activity, and the quality of work performance in relevant scenarios when using an exoskeleton.

The benefit of the Exoworkathlon approach is that it is developed as a prospective study design in which data are collected on an ongoing basis in a standardized approach. In the following pages, the new methodology and standards of Exoworkathlon will be described, as well as the development of the first so-called “Parcours.” Results and further work will be published in future articles.

## 2. Conception

Exoworkathlon aims to create a prospective data collection for the holistic evaluation of industrial exoskeletons on neutral ground in different test scenarios. In order to guarantee an exoskeleton testing that replicates real-world working scenarios and simultaneously allows an evidence-based evaluation, four Parcours have been developed and defined so far.

The research team of Fraunhofer IPA and University Stuttgart defined the chosen work-related Parcours (see Section 3), the assessment methods (see Section 4), and the working procedure. These were developed based on the experts’ many years of experience in numerous ergonomics projects (Daub et al., 2021), recent studies, as well as in close consultation and workshops with experts from the related industries for each Parcour. Furthermore, there is close cooperation with the Federal Institute for Occupational Safety and Health (BAuA) and the German Social Accident Insurance (DGUV), who approved the Parcours.

The *procedure* is standardized for each subject and Parcour and is always carried out in the same way. A working time of 1 hr is chosen to ensure that the Parcours are realistic. The task is thus physically demanding but does not exceed to high risk of overload for the participants. Each participant runs the corresponding Parcour twice - 1 hr with and 1 hr without an exoskeleton. Between the two runs, there is a break of at least 2 hrs to recover. The participants are instructed to discontinue the performance if he or she is unable or unwilling to complete the task or if problems occur during the performance (e.g., physical discomfort, malaise, or other).

Everyone receives an introduction, is fitted with the exoskeleton by an expert, and completes a test-phase with the exoskeleton before taking part in the study.

The order of exoskeleton conditions (with or without exoskeleton) is randomized to minimize the effects of fatigue that might occur from the first working phase. The recovery break is also an essential aspect to avoid these effects. The exoskeletons are randomly assigned to the subjects.

Since other laboratory studies are mainly attended by laypersons, which is to be seen as a limitation (Crea et al., 2021), inclusion criteria for the *participants* in the Exoworkathlon are that they are “young experts.” A young expert is defined as being familiar with the particular work task due to their education or professional background. In combination with real-world work scenarios, this improves the validity of the results by excluding perturbing factors from untrained participants, which is especially important when evaluating the quality (Schroeter et al., 2020). In addition, these participants can estimate much better to what extent the exoskeleton could support them in the task, considering the known real working processes.

The following exclusion criteria were defined for the study: MSDs, cardiological or neurological diseases, acute or chronic diseases, or pregnancy. The participants take part based on informed consent.

The Exoworkathlon Parcours can be performed with CE-marked upper limb and lower back *exoskeletons.* The manufacturers are invited to participate in the global prospective study of Exoworkathlon performances. The intention is not to show the pros and cons of any particular system but to set up a holistic exoskeleton evaluation study.

Since the Exoworkathlon will also take place at conferences and trade fairs, a direct evaluation of the results within a few hours is desirable. For this purpose, *analysis scripts* were created to evaluate the data directly. However, if the investigator determines that the subject is not performing the task conscientiously or is intentionally performing it incorrectly, the data will be excluded from the analysis.

## 3. Parcours

So far, the research team has defined four work scenarios that have been realistically abstracted into corresponding tasks for the Parcours. The setup and description of each Parcour are presented in detail below.

### 3.1. Parcour P1: Back-Support Exoskeletons in Logistics

Workplaces in the logistics sector are characterized by highly repetitive tasks, external weights, and non-ergonomic postures. Load manipulation or activities in static postures are known to be the most common cause of work-related musculoskeletal stress (Parent-Thirion, 2017). Here, typical tasks include lifting and carrying external weights (BAuA, Bundesanstalt für Arbeitsschutz und Arbeitsmedizin, 2019). Back-support exoskeletons could be a helpful ergonomic aid to support and relieve the lower back during this type of logistical task and have been evaluated in several studies (Hensel et al., 2018; Alemi et al., 2020; Madinei et al., 2020; Schmalz et al., 2021). In order to adapt the study conditions to the real work situation as far as possible, it is useful to study a work sequence instead of isolated movements. This enables a better transfer of the study results to the real work situation (Poliero et al., 2020).

**Figure 1.**
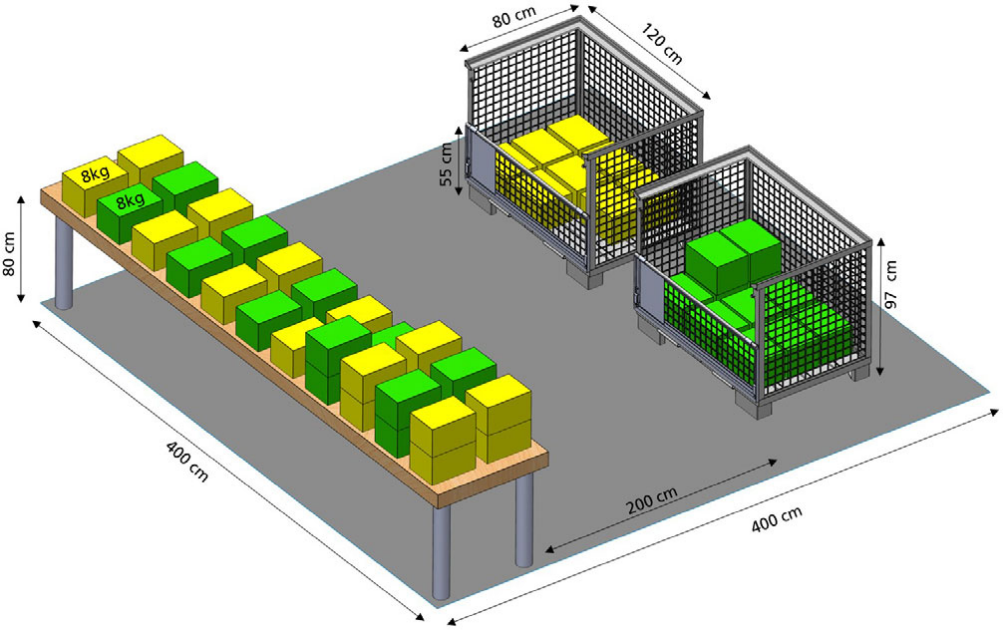
Design of P1. Table (assembly line) and two grid boxes with markings. 48× 8 kg packages (22 × 23 × 31 cm).

P1 depicts a realistic, representative task of a so-called “band cleaner” in an automotive plant. This task was selected and defined with automotive and ergonomic experts from AUDI AG to ensure a realistic workflow, walkways, weights, and heights. In this task, 8 kg packages must be picked up from a table, which represents a belt, and carried over a distance of 2 m to one of two grid boxes (see Figure 1). In logistics, different weights are common; however, this Parcour should still be feasible. Therefore, the basis for the 8 kg packages is the NIOSH Lifting Index (Waters et al., 1993) for an average person (5th to 95th percentile, Deutsches Institut für Normung (2007)) so that they are working in a medium-risk area. The packages are stacked in one of the two grid boxes according to their markings. Since sorting into different boxes is also carried out in reality, this small cognitive task was added to the Parcour to remain as close to reality as possible. The working time is based on the rhythm measured in the logistics of the automotive industry (Hensel et al., 2018). A running clock gives the participants a rough schedule to keep in order to clear all the packages within 8 min. After 8 min are completed and 48 packages are transported, the participant has a short break of 2 min. Then the participant sorts the packages back onto the table. Both tasks - sorting in and out - are present in logistics.

The process of transporting the packages into the grid boxes and back onto the belt is repeated three times so that the participants work for 1 hr.

### 3.2. Parcour P2: Exoskeletons for the Upper Limb in Automotive Assembly

Working overhead is one factor leading to work-related MSDs of the shoulder, neck, and upper extremities (Grieve and Dickerson, 2008). Upper limb exoskeletons could reduce muscular strain in the upper body, especially in the shoulder area, and relieve the physical strain on workers (Huysamen et al., 2018; Schmalz et al., 2019).

A well-known example of repetitive work at overhead height is working on an assembly line like in the automotive industry. Therefore, P2 was developed with automotive and ergonomics experts from AUDI AG (Hensel et al., 2020). Together, a realistic Parcour was developed, which depicts the typical tasks involved in underbody assembly. Both dynamic and static tasks have been defined. To ensure that the times for the tasks match those in a real work environment, duration times for each task were determined and specified via the Methods-Time Measurement (MTM) together with AUDI AG. The Parcour includes assembly and disassembly of the following tasks:

1. setting clips in a prefabricated hole (12×)
2. screwing with a cordless screwdriver into a thread (16×)
3. laying cables into nine cable holders (2×)
4. painting lines (25×, line of 390 mm).

**Figure 2.**
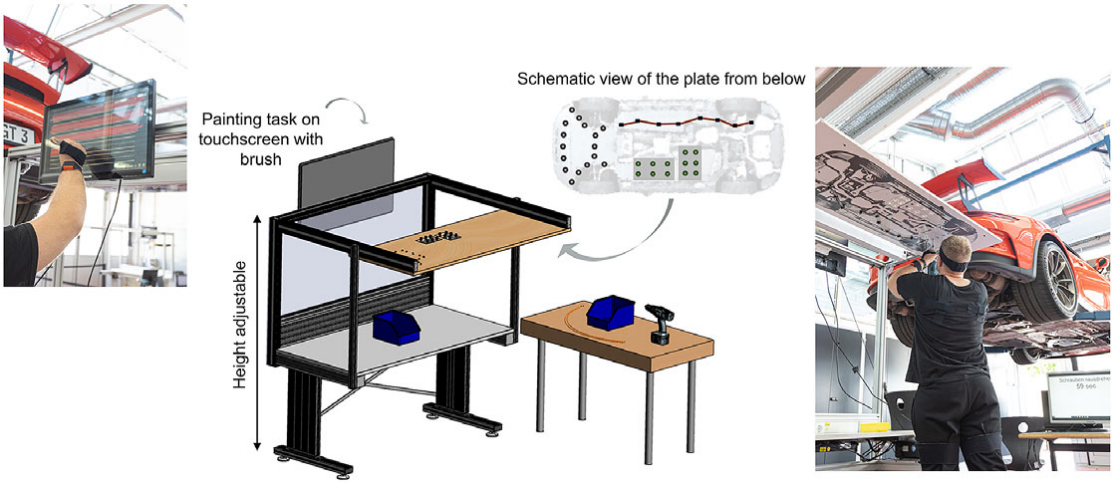
Design of P2. Height adjustable table with mounting plate with tasks in overhead height. Touchpad for the painting task at the back of the table. Material table with screwdriver, screws, clips, cables, and paintbrush with integrated touch pen, and button for time tracking.

These tasks are mapped abstractly (see Figure 2) on a test bench with the height set in an individual overhead position for each participant (body height plus hand length).

**Figure 3.**
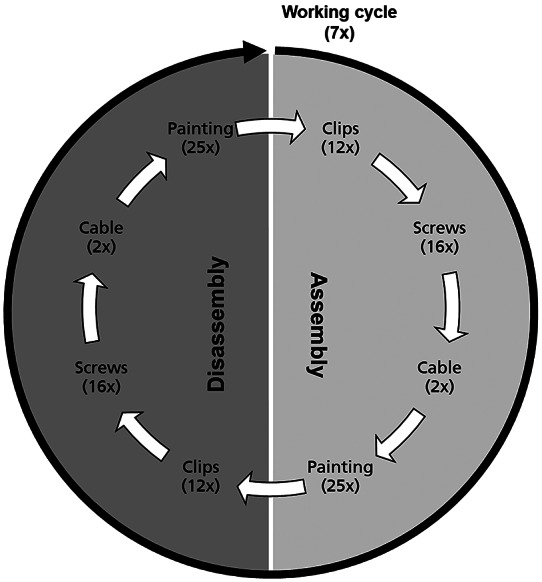
The working cycle of P2. It consists of assembly and disassembly. One whole cycle is repeated seven times within a 2 min break.

The participants have to carry out the individual tasks sequentially and repetitively according to a given time defined by MTM. Hence, the participants must keep to the working time and perform the tasks as accurately as possible, especially during painting. The countdown of the available MTM cycle time of the task is displayed on a screen. Each working time is measured by pressing a button linked to the countdown program. One working round consists of assembly and disassembly and takes 6.6 min (see Figure 3). For each task, the participant takes the needed materials from the table and goes to the worktable to perform it. When clipping and screwing, the test subject has to pick up each clip or screw separately so that the arm is lowered each time. This execution is necessary to compare the parameters (e.g., electromyography [EMG]) with and without exoskeleton. After each complete working cycle, the subjects have a 2 min break. This procedure is repeated seven times in order to add up to 1 hr of working time.

**Figure 4.**
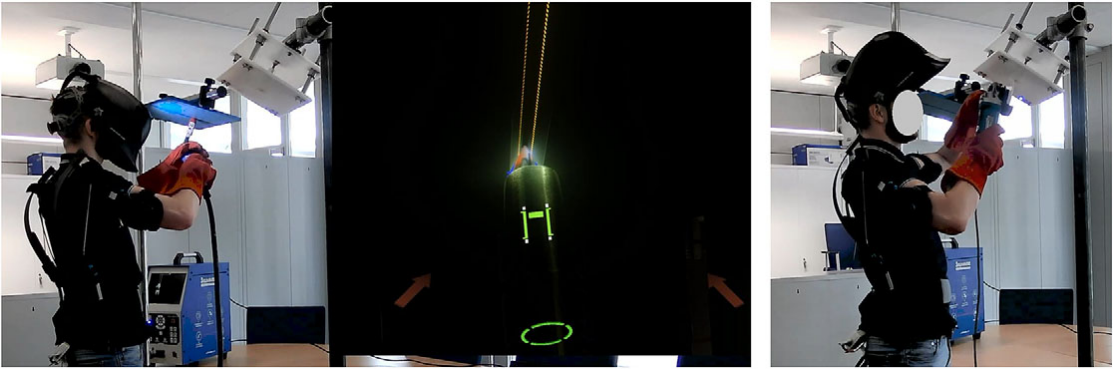
Working PE position in P3. Simulated welding in position PE and view though the AR glasses (left). Simulated grinding in PE position (right).

### 3.3. Parcour P3: Exoskeletons for the Upper Limb during Welding

The profession of the welder is very multifaceted and offers the most diverse application possibilities. Common to all workplaces is the high physical stress caused by welding equipment and protective clothing, as well as exposure to intense heat and the often noisy environment (DVS-SLV-Internationaler Schweissfachingenieur, 2022). In many cases, the respective field of application requires the adoption of constrained body positions to be able to implement the high requirements. These unnatural and extremely stressful body positions, especially when welding with raised arms or overhead, have been shown to cause disorders of the joints as well as musculoskeletal diseases in the shoulder, neck, and back area (Kadefors, 1994; Shahriyari et al., 2020). Although automation is implemented on many sides, manual welding is still essential and indispensable due to the worker’s flexibility. The challenges of this occupational field are very demanding and require a balanced combination of gross and fine motor skills that must remain high even under strong external influences. To maintain this high demand for quality continuously and at the same time reduce physical stress and thus prevent physical damage prematurely, two real welding workplaces in constrained positions were simulated to investigate the effects of exoskeletons on these activities.

The German DIN ISO 9606-1 (Deutsches Institut für Normung, 2017) served as the basis for the abstracted and simulated workplaces, making it possible to define real processes with authentic framework conditions for P3.

In order to create a safe working environment for the study that adequately reflects reality, welding simulators from the company Soldamatic were used instead of real welding equipment. These simulators are widespread and used in government and private training centers as well as in company schools. The simulators use augmented reality (AR) technology and are identical to real welding machines in shape and weight. Since this is the most widespread and economically relevant joining process, only the metal active gas welding process is represented in Parcour 3. With these modern simulators, it is possible to represent and implement arbitrarily complex workpieces.

Each work process in the welding profession also includes preparing and reworking the workpiece. For this work, angle grinders are usually used to process the applied welds layer by layer. In order to simulate this part of the work, which accounts for about 20% of the activity, as precisely as possible, the forces to be applied to the workpiece were determined in a study using force transducers. Subsequently, a device was constructed that realistically simulates the grinding task with the help of a prepared commercial angle grinder and optical force feedback.

Since there is a large number of welding positions, P3 was defined together with the SLV Nord in Hamburg, a Welding Training and Testing Institute, to determine the positions that are frequently used in everyday work and require strenuous postures. Consequently, the following positions were determined for the study according to the internationally valid standard DIN EN ISO 6947 (Deutsches Institut für Normung, 2019):

1. “PF Position” vertical uphill with the workpiece located in front of the upper body and the end position of the burner slightly below eye level.
2. “PE Position” overhead with the workpiece positioned above the head and approximately 300 mm in front of the eyes.

The experimental workflow can be divided into individual steps: “Simulated welding of a 250 mm seam” and “Simulated grinding of the weld seam.”

These performed one after the other are part of both positions (PF and PE) and repeated ten times in each (see Figure 4).

### 3.4. Parcour P4: Exoskeletons for the Upper Limb in Collaborative Tasks in Timber Construction

The timber construction industry broadly comprises two different sectors: The traditional construction method, where individual components are handled and assembled on-site, as well as off-site construction, where the prefabrication of components takes place in a controlled environment (e.g., a factory) and is then transported on-site. In both cases, workers perform collaborative tasks to handle large and/or massive components like beams and panels, facing safety risks associated with the working conditions, various activities performed, and allocating of workers’ roles. Consequently, physical stress and loads are unavoidable and have a high risk of MSDs (Kim et al., 2011; Zhu et al., 2021).

Self-observations at off-site German timber prefabrication manufacturers reveal that workers are exposed to safety hazards such as lifting heavy objects, repeating tasks, working at heights, or overhead hazards. Similar findings and recommendations regarding modular home installation environments are presented in the literature (Becker et al., 2003). Other studies reported that frequent and serial overhead work in the construction industry leads to shoulder pain in up to 30% of workers and consequent financial losses (Umer et al., 2018; EU-OSHA European Agency for Safety and Health at Work et al., 2020).

P4 was developed with construction site experts from Schwörer Haus KG to create a practical adaptation of a particular off-site work-related collaborative scenario for testing commercial shoulder support exoskeletons.

The beam is a massive and heavy element, a frequently used structural element that requires the handling assistance of two people. Another typical use case of overhead position tasks in timber construction is the installation of wooden strips. These lighter but large and difficult-to-handle elements are attachment support for posterior ceiling panel installation.

In P4, based on the modular fabrication mode for housing construction, two participants (one couple) perform two collaborative sequential assembly tasks (Parcour work cycle): positioning and fastening of a timber beam using concealed connectors and installation of wooden strips on a ceiling.

#### 3.4.1. Timber beam assembly

**Figure 5.**
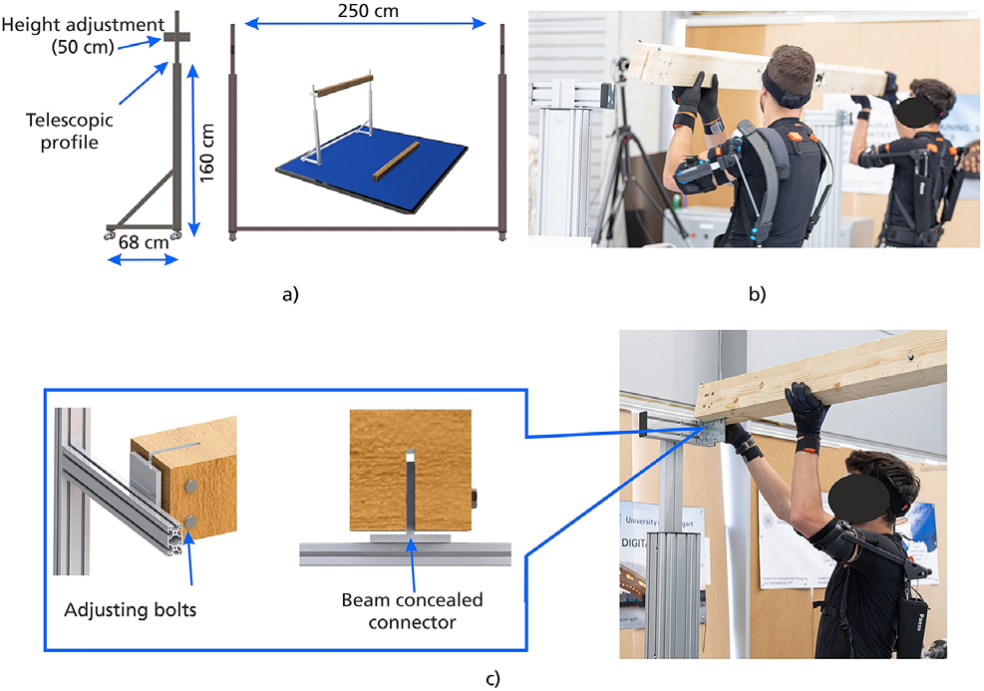
Design of P4 TBI. (a) Metallic structure (250 × 68 cm) with telescopic profile, allowing adjustable height from 160 to 210 cm. (b) The couple synchronizes movements to position the beam in the structure. (c) The beam is fastened using bolts in the concealed connectors.

Timber beam assembly (TBI) involves repeated assembling and disassembling of a timber beam from the ground to a certain height above the head so that it is fixed horizontally at that height and then lowered back to the ground. Five repetitions (assembly and disassembly) are performed to complete the task. Initially, the 2.5 m long, 13.5 kg beam is placed on the ground 1.5 m from the structure so that from this point, the couple can place the beam horizontally on the connectors on each side of the structure. The height at which the beam must be mounted varies in the different rounds (1.65 and 1.95 m). This corresponds to a height of up to 50 cm above shoulder height for men belonging to the 5th to 95th percentile of height (DIN 33402-2: Deutsches Institut für Normung, 2007). Finally, the beam is attached to the connectors with two M12 bolts and nuts on each side. Further features of the TBI scenario are shown in Figure 5.

#### 3.4.2. Wooden strips installation

**Figure 6.**
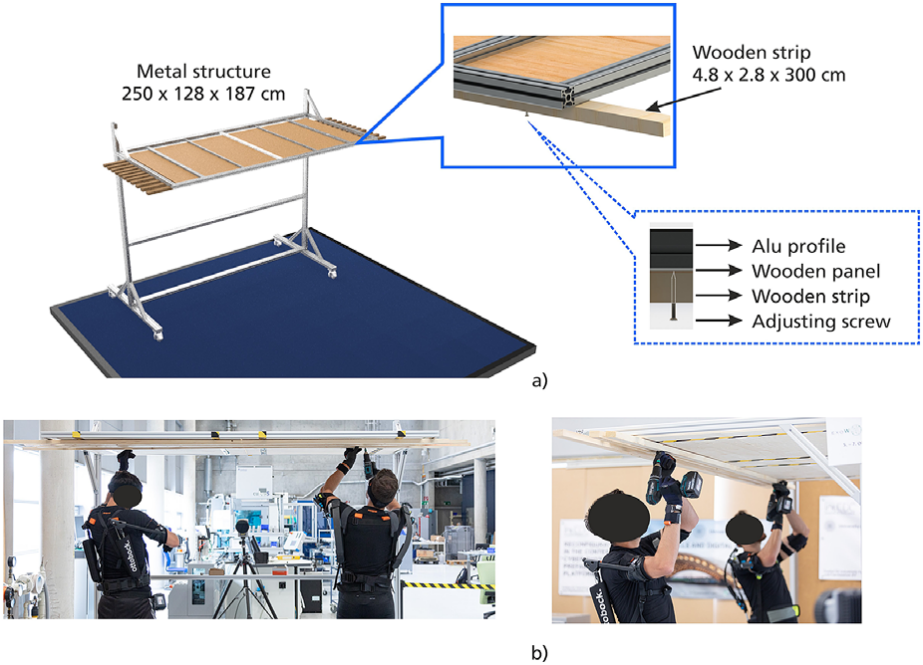
Design of P4 WSI. (a) Metallic structure (187 × 250 × 128 cm) with a roof (120 × 250 cm) made of a static wood panel and aluminum frame. (b) The participants place and fasten ten strips (4.8 x 2.8 x 300 cm).

Wooden strips installation (WSI) consists of placing 10 units of 3 m long wooden strips on a metallic structure ceiling. The strips weight varies from 1.4 to 1.8 kg. The couple coordinates their movements to place each strip horizontally and adjust it to the wooden ceiling panel with six screws (three/person). The panel is at the height of 1.9 m. One repetition is performed to complete the task. Figure 6 shows more features of the WSI scenario.

The work cycle (TBI + WSI) lasts 1 hr when both tasks are performed three times.

## 4. Assessments

For an intra-individual anonymous comparison, the data are collected through various evaluations in the Parcours.

The expert team defined the assessments based on standard methods in previous studies (Crea et al., 2021; De Bock et al., 2022). Even though the assessment methods in these studies differ greatly, there are certain parallels. Mostly, similar parameters were used to define the workload. Especially, subjective user feedback is an assessment that is usually used. Furthermore, EMG and metabolic costs are often included to assess objective physical parameters. Besides physiological parameters, quality aspects are interesting objective parameters that can show advantages or disadvantages of exoskeletons in terms of duration times or work output quality. On this basis, subjective and objective assessments were defined (see Table 1 and deeper explanations below).

**Table 1.** Assessments of Exoworkathlon

Assessments
*Subjective user feedback*
Effort of the task (0 none – 10 maximum)
Body discomfort scale (1 nothing – 8 extremely hard)
System Usability Scale (1 strongly disagree – 5 strongly agree)
*Electromyography* (Mean muscle activity [%MVC] in combination with the movement)
M. erector spinae activity (during forward bending, in back-Parcours)
M. deltoideus clavicularis and acromialis activities (during overhead work, dominant arm, in overhead-Parcours)
*Cardiovascular load*
Heart rate (beats per minute [BPM])
Hemodynamic and electrical cardiac conduction parameters (by use of impedance cardiography)
*Quality (Parcour specific)*
Duration of the working time
Accuracy in overhead painting task (error score: counts pixel overpainting the given line)
Assessment of weld seam quality (rating of welding on a scale between 0 and 100% by AR simulator)

*Abbreviation:* MVC: maximum voluntary isometric contraction.

In principle, all assessments could be applied to all Parcours, except the task-specific quality assessments.

### 4.1. Subjective User Feedback

The questionnaire acquires subjective user feedback from the participants. The perceived exertion of the performed task is queried via the established BORG-CR10 Scale (Borg, 2004). In addition, a Body Chart by Corlett and Bishop (1976) is used for specific stress perception ratings for the individual body areas.

Perceived exertion and body areas stress are queried for both the tests with and without the exoskeleton. The questionnaire should be filled in after each completed round to investigate a change over time. The rounds depend on the task set for each Parcour (see definition in Parcour description). After using the exoskeleton, an additional questionnaire is filled in to evaluate the systems. This includes the System Usability Scale (Brooke, 1996) and additional questions on the feeling of safety and discomfort created by Fraunhofer IPA and University Tuebingen.

### 4.2. Quality

In P2 and P3, the quality of work can be measured.

In P2, the time required for the tasks and the accuracy in executing the tasks can be assessed. The times given for each task are based on MTM, which plays an essential role in the planning of manual operations in the industry. The times were defined by an ergonomics expert from the automotive industry. Working time is measured by a scripted program linked to a button. After completing the respective task, the participants have to press the button and the required working time is measured and saved.

A second assessment of quality in P2 is the error score of the painting task. This captures the accuracy in painting and is measured when painting over a defined line by an app. If the line is painted over, the error score increases and is saved as the average error score for one cycle.

In P3, the quality of the work performed is evaluated via the Soldamatic welding simulation software. Each weld seam is scored on a scale from 0 to 100% by the Soldamatic software based on five parameters. Work angle, travel angle, contact to work distance, travel speed, and aim influence the weld seam quality. Based on these parameters, each seam received an individual and overall evaluation and can thus be compared with each other.

In the results, the quality is shown as the difference between the execution with and without exoskeleton.

### 4.3. Electromyography

EMG is a standard tool to record muscle activity during work in combination with movements. Depending on the most used and stressed muscles in the selected Parcour and the supported body area of the exoskeleton, the muscles to be recorded are specially selected for the Parcours. It is well known that it is always helpful to examine several muscles to analyze load redistribution or compensatory movements further. Therefore, additional EMG sensors can be added anytime for deeper analyses. In this setup, however, the activity’s corresponding main muscles are considered first to check the exoskeletons’ main expected effect. Furthermore, for evaluation, the activity of the muscles in relevant, specific body positions was considered, and a range of motion was defined in each case in which the EMG data should be included into the analysis: For the upper body Parcours, the overhead working height is defined as the threshold from which EMG data were included. For the lower body Parcour, the forward bending when picking up the packages from the grid is defined as the threshold. These movements are recorded with a motion capture system combined with an EMG System due to the motion-dependent EMG analysis.

The selected muscles are localized by specific tension and palpation. Skin preparation and placement of the sensors are according to the SENIAM (1999) guidelines.

To normalize the muscle activity of the recorded muscle, the reference value needs to be obtained by performing maximum voluntary isometric contraction (MVC) in the functional position of the respective muscles before starting the work. The normalized muscle activity (%MVC) is used to determine the mean value across all subjects with and without exoskeleton as a parameter of the EMG. The EMG data are analyzed over time per round and in P2 on the individual tasks.

### 4.4. Cardiovascular Load

A commercially available smartwatch is used to measure the heart rate and also provides a calculated oxygen consumption. Heart rate is a good indicator of physiological load, as it regulates the heart’s performance as a factor for cardiac output (Klinke and Silbernagl, 2003).

In order to determine other cardiovascular effects, an impedance cardiograph is used. Hemodynamics and cardiac conduction that describe the cardiovascular system are recorded. Therefore, two pairs of electrodes are applied to each subject’s neck and thorax. The interface can be worn on the hip. This method is typically used for patient monitoring in intensive care units. Previously performed measurements indicate that the changes in hemodynamics and electrical cardiac conduction could be used to evaluate exoskeletons, as they provide more detailed insight into cardiovascular load (Stegemann, 1991).

## 5. Conclusion and Outlook

Research methods and evaluation of exoskeletons have several shortcomings despite the studies conducted to date. To understand these effects, an evaluation of the systems in real work situations is necessary. Furthermore, standardized test procedures are essential to acquire a large and comparable data pool.

To this end, this article introduced a modular study design to prospectively collect data and strengthen the exchange between developers, researchers, and end-users to advance the young exoskeleton industry jointly. This should motivate further research groups to stick to this study protocol to add further data sets in a continuous, multicentric prospective study approach.

Exoworkathlon makes it feasible to test and evaluate exoskeletons under working conditions that are as close to reality as possible in specially developed Parcours. This provides the opportunity for intraindividual comparisons of exoskeleton users and for generating study data per exoskeleton type.

The Parcours of Exoworkathlon need to fulfill the following crucial aspects. They must be defined in cooperation with experts from the corresponding industry and health sectors like the DGUV and BAuA to be realistic and feasible as well as a relevant task for exoskeleton use. Furthermore, the Parcours must be completed by professional workers for 1 hr with and 1 hr without an exoskeleton.

Based on these criteria, four Parcours, related assessments, and standardized procedures have been developed and presented in this article. In addition, the Parcours are to be extended to further relevant modular use cases and related assessment methods with interested expert partners from industries and occupational health. This methodology will be further conducted as prospective true worker studies under the above aspects with exoskeleton manufacturers and end-user industries to evaluate and ideally strengthen the evidence of exoskeleton use benefits. Exoworkathlon is carried out in the industry, at trade fairs, conferences, and professional schools. During those implementations, an active exchange among the test persons, end-users, scientists, and manufacturers is possible so that the feedback and exoskeleton potentials can be discussed together.

The participated exoskeleton manufacturers can receive the results of their system to compare those data with the overall anonymous evaluation of all systems and thus identify potential areas for improvement.

The prospective data collection results will be published in further research papers and updated on an online platform (www.exoworkathlon.de) to keep the ongoing study results freely accessible for everyone to follow.

## Data Availability

The data that support the findings of this study are available from the corresponding author upon reasonable request.
